# Symptoms and impacts in anemia of chronic kidney disease

**DOI:** 10.1186/s41687-020-00215-8

**Published:** 2020-07-29

**Authors:** Susan D. Mathias, Steven I. Blum, Vanja Sikirica, Kirsten L. Johansen, Hilary H. Colwell, Tony Okoro

**Affiliations:** 1 Health Outcomes Solutions, PO Box 2343, Winter Park, FL 32790 USA; 2 GlaxoSmithKline, 1250 S. Collegeville Road, Collegeville, PA 19426 USA; 3 Currently at Bristol-Myers Squibb, Lawrenceville, NJ USA; 4 Currently at Pfizer, Collegeville, PA USA; 5 Hennepin Healthcare, University of Minnesota, 730 South 8th Street, Minneapolis, MN 55415 USA

**Keywords:** Patient-reported outcomes, Semi-structured interview, Concept elicitation, Cognitive debriefing, Anemia, Chronic kidney disease

## Abstract

**Background:**

Anemia is a frequent complication of chronic kidney disease (CKD) that negatively affects patients’ health-related quality of life.

**Methods:**

We conducted qualitative concept elicitation (CE) and cognitive debriefing (CD) interviews to assess the frequency, duration, and severity of symptoms and impacts associated with anemia of CKD and to facilitate the development of a new patient-reported outcome (PRO) measure. We interviewed 36 patients with CKD and hemoglobin levels ≥8.0 to <12.0 g/dL using a semi-structured interview guide developed specifically for this study until saturation was reached. We used MAXQDA to perform qualitative analysis of interview transcripts to determine the most relevant symptoms and impacts (based on the frequency of concept mentions) experienced by participants.

**Results:**

Most participants had stage 4/5 CKD (81%) and were being treated with an erythropoietin stimulating agent (69%). Spontaneously reported symptoms included feeling tired (79%), shortness of breath (39%), and weak/lacking strength (36%). We developed the Chronic Kidney Disease and Anemia Questionnaire (CKD-AQ), which includes 23 items assessing frequency and severity of the most relevant symptoms and impacts identified by patients with anemia of CKD. The CD interviews confirmed the clarity and relevance of the concepts identified in the CE phase.

**Conclusion:**

The CKD-AQ is a novel PRO measure that captures the frequency and severity of the most relevant symptoms and impacts associated with anemia of CKD. Future studies will evaluate its psychometric properties and its potential utility in anemia management.

## Background

Anemia is a frequent complication of chronic kidney disease (CKD) that increases in prevalence and severity as kidney function declines [1, 2]. Common symptoms of anemia of CKD include low energy, fatigue, and decreased physical function, which can negatively affect patients’ health-related quality of life (HRQoL) [2]. Although clinical laboratory values like hemoglobin have been used to monitor patients’ anemia, such measures do not capture how patients feel and function. For optimal patient care, additional tools to assess the symptoms of anemia of CKD and their effect on patient well-being are needed.

Patient-reported outcome (PRO) measures capture concepts such as the symptoms and/or impacts of a disease from the patient’s perspective [3–5]. They are increasingly being used as part of the evaluation of new treatment options or when comparing the efficacy of different treatments [3, 6]. Over the past several years, various agencies and organizations, including the US Food and Drug Administration (FDA), International Society of Pharmacoeconomic and Outcomes Research (ISPOR), and European Medicines Agency (EMA), have developed best practices to ensure appropriate content validity and psychometric properties of PRO measures [6–10]. These recommendations include defining the disease and target patient population (context of use) in whom the measure will be used and specifically obtaining patient input on relevant symptoms and impacts (concepts of interest). Additionally, cognitive interviews can help to confirm clarity and relevance to patients of the concepts being evaluated by the PRO measure. Well-developed, validated PRO measures may also improve clinical care by helping clinicians understand patients’ symptoms [3–5] and could potentially facilitate communication between patients and physicians and improve patients’ satisfaction with treatment [5, 11].

PRO measures have been developed for patients with CKD and separately for patients with anemia; however, the relevance of these measures to patients with anemia of CKD has not been fully determined [12–16]. A PRO measure, developed in accordance with best practices, could provide meaningful information about symptoms and impacts experienced by patients with anemia of CKD [6, 9, 10]. The objectives of this study were to conduct qualitative interviews with CKD patients who experience anemia to better understand the symptoms associated with anemia of CKD and their effect on patients’ lives and to use this information to develop a novel disease-specific PRO measure.

## Methods

### Study flow

**Fig. 1:**
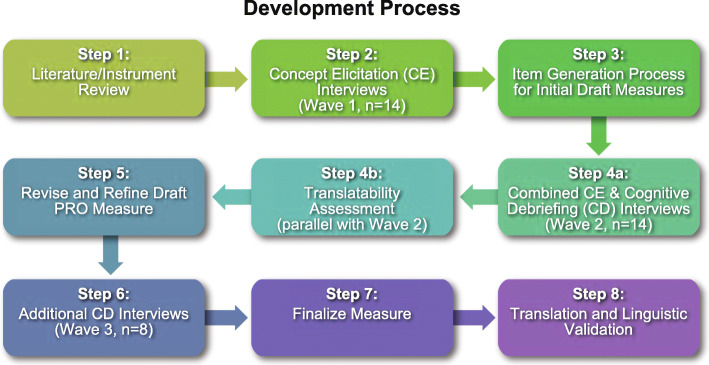
PRO Measure Development Process. Overview of the development process for a novel questionnaire to evaluate the symptoms and impacts of anemia of CKD

Following FDA, ISPOR, and EMA best-practice guidelines on PRO development and measurement [6–10], we used an iterative approach to conduct patient interviews and develop and evaluate the PRO measure (Fig. 1). Data collection and analysis were also done following FDA, ISPOR, and EMA best-practice guidelines. The various steps in the development process are as follows:

#### Step 1

We conducted a targeted review of literature articles published between January 2006 and March 2016 using the following search string: (CKD OR “Chronic Kidney Disease”) AND (symptoms OR anemia OR fatigue OR energy OR cognition OR memory) AND questionnaire, to identify concepts to explore during subsequent qualitative patient interviews, and to identify existing CKD and anemia-specific PRO measures. Our targeted literature review identified 5 existing PRO measures (The Kidney Disease Quality of Life Instrument [KDOQOL], the Functional Assessment of Cancer Therapy – Anemia [FACT-an], The Fatigue Assessment Scale [FAS], Patient-Reported Outcome Measurement Information System Fatigue [PROMIS Fatigue], and Dialysis Symptom Inventory [DSI]) with potential relevance in the anemia of CKD population (Item S3 and Table S2) [12–16]; however, none of these specifically examined anemia in the context of CKD, nor were they developed with input from patients with anemia of CKD. Consequently, we decided to conduct qualitative interviews and to potentially develop a new measure specific for this patient population.

#### Step 2

We developed a semi-structured interview guide, which included open-ended questions designed to facilitate discussion, with additional probing to further explore concepts as needed. We made minor revisions to the interview guide after the first few interviews. Qualitative concept elicitation (CE) interviews (*N* = 14) were conducted in accordance with good research practices [6] by telephone (Wave 1) to identify relevant symptoms and impacts as reported by patients with anemia of CKD. All interviews were conducted in English by interviewers experienced in conducting qualitative interviews in a manner that encouraged participant engagement and open communication. Interviewer characteristics and sample interview questions are summarized in items S1 and S2 and Table S1.

#### Step 3

An initial item generation process resulted in the development of 2 PRO measures, a 23-item daily symptom diary, and an 18-item weekly impact questionnaire, based on a thematic analysis of the interview transcripts to identify the most relevant symptoms and impacts and the frequency of symptoms reported by study participants during the CE interviews. To the extent possible, questions were constructed using language expressed by participants.

#### Step 4a

A second wave of interviews (*N* = 14) was conducted in a separate set of participants to further explore concepts of interest in patients with anemia of CKD and to assess the content, clarity, and relevance of the 2 draft PRO measures developed in Step 3. These in-person interviews combined CE and cognitive debriefing (CD) components [10] and were conducted one-on-one at either the dialysis center or nephrology clinic. Participants were first asked an abbreviated set of the CE questions from the initial interviews and then asked to provide feedback on the draft measures. The draft PRO measures were updated as interviews were conducted. All revisions to the measures and the rationale for revising were documented using an item-tracking matrix. Because interview time was limited (~ 60 min), not all participants were asked all interview questions.

#### Step 4b

In parallel to Wave 2 interviews (Step 4a), a translatability assessment was conducted by experienced translators in Hindi, Russian, and Spanish to assess the feasibility of translating the draft items into other languages for use in global studies [17]. The translatability assessment was completed to determine where difficulties would be encountered in subsequent translation efforts for the new PRO measures. The languages were selected because they represent diverse language families spoken by large populations.

#### Step 5

Based on results from the CD interviews (Wave 2) and input from the research team, it was determined that a 7-day recall period would increase the practicality for using the new PRO measures in clinical trials. As such, the content of the 2 draft PRO measures was combined into a single questionnaire, with a 7-day recall period for all symptoms (except for bruising, which uses a 1-month recall period).

#### Step 6

An additional wave of CD interviews (*N* = 8) (Wave 3) [10] was conducted via telephone to obtain feedback on the content, relevance, and clarity of the newly combined PRO measure and to confirm the revisions in Step 5, including the relevance of the 7-day recall period for the items previously included in the daily diary.

#### Step 7

Following completion of Wave 3 interviews (Step 6), the content of the PRO measure was finalized.

#### Step 8

The measure was translated into additional languages. All translations underwent either full linguistic validation, including dual forward translations and dual backward translations or linguistics review. All translations were subsequently reviewed by a clinician and underwent cognitive debriefing with 5 patients, as well as proofreading and quality control (QC) steps throughout. The translation process included full linguistic validation interviews.

### Study population

Human subjects research approval for this project was provided by an independent scientific review committee (The Copernicus Group, Cary, NC). All participants provided informed consent before enrolling in the study.

Study participants were recruited from Fresenius Medical Care North America (Research by Design site in Evergreen, IL) and DaVita Clinical Research network practices in the United States via telephone and in person. Recruitment was designed to select a diverse range of patients with anemia of CKD rather than replicating real-world demographics. Therefore, an effort was made to diversify recruitment, including a heterogeneous sample across stages of CKD and type of dialysis, sex, age, educational levels, and race [6]. Although sampling relied on a convenience sample, a recruitment target was used whereby ≥3 patients were sought in each subgroup of interest, including patients treated with erythropoietin-stimulating agents (ESAs), those receiving intravenous iron, treatment naïve patients, dialysis-dependent patients, and those not currently receiving dialysis.

To be included in the study, participants were required to be US residents, aged ≥18 years, and have a confirmed diagnosis of CKD. All study participants were required to have hemoglobin (Hb) levels ≥8.0 g/dL and < 12.0 g/dL. However, an effort was made to include patients with both low and high Hb levels, with low Hb defined as 8.0 to 9.9 g/dL in dialysis-dependent patients and 8.0 to 8.9 g/dL in non-dialysis patients and high Hb defined as 10.0 to 11.0 g/dL in dialysis-dependent patients and ≥9.0 g/dL in non-dialysis patients. Participants also needed to speak and read English fluently, provide consent to participate, and be willing to participate in a single audiotaped interview (in person or by telephone) of approximately 60 min.

Patients not on dialysis were eligible regardless of whether they were being treated with an ESA. However, any change in ESA use (initiation or discontinuation) must not have occurred within the past 12 weeks. Patients receiving dialysis were eligible if they were currently receiving an ESA for ≥12 weeks and were on dialysis for ≥12 weeks. Patients who had initiated dialysis within the past 4 weeks were also eligible if they were not currently receiving an ESA. Patients undergoing hemodialysis were required to receive dialysis ≥2 times weekly. Patients undergoing peritoneal dialysis had to be on daily dialysis to be included in the final round of CD interviews (Wave 3).

Patients with medical or psychiatric conditions or those being treated for a condition that resulted in a cognitive or other (e.g., visual, hearing) impairment that would interfere with study participation (based on the investigator’s opinion) were excluded.

### Analysis

All interviews were audio recorded and transcribed. All data from the interviews was then coded using MAXQDA (version 11.1.2). A code book was developed iteratively to categorize concepts of interest from the interviews and included descriptions and examples for each code to ensure consistency across coders. Each transcript was coded by 1 coder, then reviewed, summarized, and analyzed by a second coder for accuracy. A saturation table was developed to document table to document the point at which no new concepts were mentioned by subsequent participants for each symptom mentioned during Wave 1 and the CE portion of Wave 2 of the study. Analyses for subgroups were also conducted of interest including dialysis vs non-dialysis patients, hemodialysis vs peritoneal dialysis patients, and patients with Hb level <10.0 g/dL vs those with >10.0 g/dL. Subgroup analyses were descriptive only, and no formal statistical testing was done.

## Results

### Demographic and clinical characteristics

A total of 36 participants were interviewed: 14 participated in the initial CE telephone interviews (Wave 1), 14 participated in combined CE/CD interviews (Wave 2), and a further 8 participated in an additional set of CD interviews (Wave 3) over the telephone. Demographic and clinical characteristics are summarized in Table 1.

**Table 1:** Patient Demographic and Clinical Characteristics

	Wave 1 CE Interviews (*n* = 14)	Wave 2 Combined CE/CD Interviews (*n* = 14)	Wave 3 CD Interviews (*n* = 8)	All Participants (*n* = 36)
Age, years, mean ± SD (range)	65 ± 17.6 (30–83)	55 ± 15.8 (32–82)	71.4 ± 14.6 (48–88)	62.7 ± 17.1 (30–88)
Gender, n (%)				
Male	4 (29)	3 (21)	3 (38)	10 (28)
Female	10 (71)	11 (79)	5 (62)	26 (72)
Race/Ethnicity, n (%)				
White	7 (50)	2 (14)	0	9 (25)
Black	5 (36)	9 (64)	8 (100)	22 (61)
Hispanic	2 (14)	3 (21)	0	5 (14)
Education				
Less than HS	0	2 (14)	0	2 (6)
HS diploma	4 (29)	4 (29)	1 (13)	9 (25)
Some college	10 (71)	6 (43)	5 (63)	21 (58)
College degree	0	2 (14)	1 (13)	3 (8)
Professional or advanced degree	0	0	1 (13)	1 (3)
CKD stage, n (%)				
Stage 3a	1 (7)	0	0	1 (3)
Stage 3b	2 (14)	1 (7)	3 (38)	6 (17)
Stage 4	3 (21)	5 (36)	2 (25)	10 (28)
Stage 5 (not on dialysis)	0	0	2 (25)	2 (6)
Stage 5 (on dialysis)	8 (57)	8 (57)	1 (13)	17 (47)
Comorbid conditions, n (%)				
Myocardial infarction	1 (7)	2 (14)	0	3 (8)
Stroke/transient ischemic attack	0	1 (7)	1 (13)	1 (6)
Arterial hypertension	7 (50)	6 (43)	8 (100)	21 (58)
Diabetes mellitus	5 (36)	6 (43)	4 (50)	15 (42)
Cardiovascular disease	1 (7)	0	1 (4)	5 (14)
Congestive heart failure	2 (14)	7 (50)	2 (25)	11 (31)
Lung disease	2 (14)	3 (21)	0	5 (14)
Receiving dialysis, n (%)				
Hemodialysis	5 (36)	8 (57)	1 (13)	14 (39)
Peritoneal dialysis	3 (21)	3 (21)	0	6 (17)
No	6 (43)	3 (21)	7 (87)	16 (44)
Treatment with ESA, n (%)				
Yes	11 (79)	12 (86)	2 (25)	25 (69)
No	3 (21)	2 (14)	6 (75)	11 (31)
Hemoglobin, mean ± SD (range, g/dL)	9.8 ± 0.9 (8.7–11.5)	9.5 ± 0.9 (7.2–10.4)	9.1 ± 0.7 (8.0–9.7)	9.5 ± 0.9 (7.2–11.5)
Iron treatment, n (%)				
Intravenous	6 (43)	8 (57)	1 (12)	15 (42)
Oral	3 (21)	2 (14)	7 (88)	5 (14)

Abbreviations: *CD* Cognitive debriefing, *CE* Concept elicitation, *CKD* Chronic kidney disease, *ESA* Erythropoietin-stimulating agents, *HS* High school, *SD* Standard deviation

Fifty-three percent of participants had stage 5 CKD. Of these, 89% were on dialysis. Overall, in the CE and combined CE/CD interviews, 57% of participants were on dialysis. To ensure representativeness of concepts within the non-dialysis population, fewer participants in the final CD group had stage 5 CKD (38%, *n* = 3), with only 1 of these 3 participants on dialysis.

### Symptoms of anemia of CKD

The frequency with which concepts were reported spontaneously and after probing is summarized in Table 2. Feeling tired (79%, *n* = 22/28), shortness of breath (39%, *n* = 11/28), and feeling weak/lacking strength (36%, *n* = 10/28) were reported spontaneously most frequently (Table 2). Feeling tired (83%, *n* = 5/6) and shortness of breath (83%, n = 5/6) were also frequently reported after probing, as well as gastrointestinal (GI) symptoms (60%, *n* = 6/10) and difficulty sleeping (50%, *n* = 12/24). Difficulty remembering things (50%, *n* = 12/24), difficulty concentrating (38%, *n* = 9/24), and restless legs (36%, *n* = 5/14) were only reported after probing. Saturation, or the point when no new information is elicited, was reached for all concepts by the 25th interview. However, additional interviews were conducted to confirm saturation, and no new concepts were mentioned except “craving ice.”

**Table 2:** Symptoms Reported During Concept Elicitation (Waves 1 and 2)

Symptom	Spontaneously Reported n/N (%)^a^	Reported After Probing n/N (%)^b^	Total Mentions of a Concept	
			Proportion spontaneous n/N (%)^c^	Proportion after probing n/N (%)^d^
Feeling tired	22/28 (79)	5/6 (83)	22/27 (81)	5/27 (19)
Shortness of breath	11/28 (39)	5/6 (83)	11/16 (69)	5/16 (31)
Feeling weak/lacking strength	10/28 (36)	0/0	10/10 (100)	0/10 (0)
Gastrointestinal symptoms	5/28 (18)	6/10 (60)	5/11 (45)	6/11 (55)
Difficulty sleeping	4/28 (14)	6/12 (50)	4/10 (40)	6/10 (60)
Difficulty remembering things	0/28 (0)	12/24 (50)	0/12 (0)	12/12 (100)
Difficulty concentrating	0/28 (0)	9/24 (38)	0/9 (0)	9/9 (100)
Restless legs	0/28 (0)	5/14 (36)	0/5 (0)	5/5 (100)

^a^n represents the total number of participants who spontaneously mentioned the symptom and N represents all patients who participated in the concept elicitation interviews

^b^n represents the number of participants who reported experiencing the symptom and N represents the number of participants probed regarding the symptom

^c^n represents spontaneous mentions of the symptom and N represents the total mentions of the symptom (spontaneous and probed)

^d^n represents mentions of the symptom after probing and N represents the total mentions of the symptom (spontaneous and probed)

### Effect of symptoms on patients’ daily lives

Ninety-five percent of participants (*n* = 19/20) reported that their daily activity was affected, 91% (*n* = 21/23) that physical functioning was affected, 63% (*n* = 10/16) that they were affected emotionally, and 45% (*n* = 10/22) that anemia of CKD affected their social functioning (Table 3). The symptom with the largest effect on daily activities varied by participant; of the 13 participants asked, 6 (46%) indicated tiredness, 2 (15%) shortness of breath, 2 (15%) no symptoms, and 1 each (8%) feeling weak/shaky, difficulty sleeping, and constipation. Likewise, participants differed in which symptom they found most bothersome and most difficult to manage. Of the 11 participants asked, 7 (64%) indicated tiredness was the most bothersome, 3 (27%) shortness of breath, and 2 (18%) difficulty sleeping. Three of the 11 participants asked (27%) indicated that being tired was the most difficult symptom to manage, 2 (18%) shortness of breath, 2 (18%) difficulty sleeping, and 1 each (9%) for dry/itchy skin, feeling weak/shaky, and constipation. Representative quotes about symptoms and their impacts are shown in Table 4.

**Table 3:** Impacts Reported

Effect	Total Mentions of Concept n/N (%)^a^
Interference with daily activities	19/20 (95)
Physical impact	21/23 (91)
Emotional impact	10/16 (63)
Social impact	10/22 (45)

^a^n represents total mentions (spontaneous and probed) of the symptom and N represents the sum of patients who reported the symptom (spontaneous and probed) plus the number of patients probed with regard to the symptom who indicated not experiencing the symptom

**Table 4:** Representative Quotes of Symptom and Impact Concepts

Feeling Weak or Lacking **Strength**	**Feeling Tired**	**Shortness of Breath**
Subject 1 “I don’t have the strength I used to have and it tires me.”	Subject 1 “The worst thing is the fact that I’m very tired. Why? Because I can’t do the things I’d like to do because I’m too tired.”	Subject 2 “The shortness of breath makes you tired and I have to sit down and catch my breath. Like now I’m talking too much so my breath is starting to get heavy.”
**Difficulty Remembering Things**	**Difficulty Sleeping**	**Gastrointestinal Symptoms**
Subject 3 “I don’t remember things as well as I used to. I write down a lot of things. I do write a lot of things down because I do forget.”	Subject 4 “That changes because there are nights I cannot fall asleep at all. I try to go to bed, do everything and nothing happens. Sometimes it be that way and then there are times where I could just doze off and keep waking up.”	Subject 5 “Constant constipation. It never goes away because number one, I’m not getting enough exercise and number two, I don’t drink enough fluid.”
**Interference with Daily Activities**	**Emotional Impact**	**Social Impact**
Subject 5 “There’s a lot of things like cooking, cleaning, just ordinary things, being able to go to the bathroom, a lot of things that people take for granted that I can’t do anymore.”	Subject 6 “Really like a burden on my family. It saddens me.”	Subject 7 “Sometimes, but for the most part no. I feel better when I’m with friends or relatives or even talking to neighbors, but like I said there’s times I don’t feel like making a call because I just don’t feel like talking on the phone. I just put it off for another day.”

Some participants had difficulty attributing their symptoms to anemia vs CKD or other health conditions (Table 5). Whereas 80% of participants attributed feeling weak/lack of strength to anemia, only 44%, 33%, and 19% identified being tired, difficulty concentrating, and shortness of breath as being due to anemia, respectively. For the remaining commonly reported symptoms (difficulty remembering, difficulty sleeping, restless legs, and GI symptoms), none attributed them to anemia.

**Table 5:** Attribution of Symptoms

Symptom	Attributed to Anemia n/N (%)	Attributed to CKD n/N (%)	Attributed to Something Else n/N (%)	Did Not Know Cause n/N (%)
Feeling weak/lack of strength^a^	4/5 (80)	1/5 (20)	1/5 (20)^b^	1/5 (20)
Feeling tired	12/27 (44)	1/27 (4)	4/27 (15)^c^	10/27 (37)
Difficulty concentrating	2/6 (33)	0/6 (0)	0/6 (0)	4/6 (67)
Shortness of breath	3/16 (19)	0/16 (0)	5/16 (31)	8/16 (50)
Difficulty remembering	0/9 (0)	1/9 (11)	0/9 (0)	8/9 (89)
Difficulty sleeping	0/9 (0)	0/9 (0)	1/9 (11)^d^	8/9 (89)
Restless legs^a^	0/5 (0)	1/5 (20)^d^	1/5 (20)^e^	4/5 (80)
GI symptoms^a^	0/10 (0)	4/10 (40)^e^	1/10 (10)^f^	7/10 (70)

Abbreviations: *CKD* Chronic kidney disease, *GI* Gastrointestinal

^a^The number of responses is greater than the number of participants asked the question because some participants attributed a symptom to more than 1 thing

^b^One participant attributed feeling weak/lack of strength to dialysis

^c^Three participants attributed feeling tired to too much physical exertion and 1 participant to depression

^d^One participant attributed difficulty sleeping to coughing

^e^One participant attributed restless legs to dialysis

^f^One participant attributed GI symptoms to dialysis

### Subgroup analyses

Results were similar across subgroups with a few exceptions. Specifically, patients not on dialysis were more likely to have difficulty sleeping (71%, *n* = 5/7 vs 56%, *n* = 5/9), whereas patients on dialysis were more likely to experience difficulty concentrating (47%, *n* = 7/15 vs 22%, *n* = 2/9). Additionally, 83% (*n* = 5/6) of those with Hb ≥10.0 g/dL reported difficulty sleeping compared with 50% (*n* = 5/10) of those with Hb <10.0 g/dL.

### Development and refinement of the PRO measures

During the initial cognitive debriefing interviews (Step 4a), participants were generally able to accurately paraphrase each item in each measure. However, several participants had difficulty understanding the item assessing “restless legs.” Based on participants’ feedback and the translatability assessment, we made minor revisions to 13 of 23 questions in the daily symptom questionnaire and 2 of 18 questions in the weekly impact questionnaire.

Further revisions to the PRO measures were made based on study team review. Items not directly related to anemia and concepts potentially redundant with existing generic HRQoL PRO questionnaires, 36-Item Short Form Survey (SF-36), and EuroQol-5D (EQ-5D) were removed, as these measures are typically used in clinical research and may be used together with the novel PRO measure. Based on clinician input regarding chest pain as a potential symptom of anemia and because patients with CKD have higher risk of cardiovascular disease, 2 questions on symptoms of chest pain were added. Finally, the recall period for all items was changed to the “past 7 days” (except a 1-month recall period for bruising) based on participant responses, indicating a 7-day recall period was acceptable. The abbreviated content and uniform recall period allowed the 2 questionnaires to be merged into a single PRO measure.

The revised PRO measure was evaluated in a final series of 8 CD interviews (Wave 3). Generally, participants correctly paraphrased each item (range: 88% to 100%), found the questions to be clear (range: 67% to 100%), and found the 7-day recall period acceptable. Minor revisions were made to 2 questions to improve clarity.

### CKD and anemia questionnaire

**Fig. 2:**
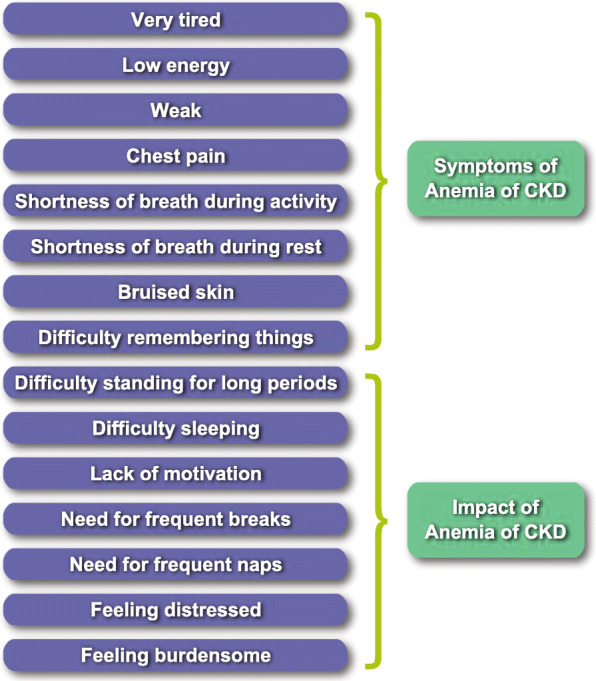
Conceptual Framework

The final CKD and Anemia Questionnaire (CKD-AQ) contains 23 items covering relevant symptoms and impacts associated with anemia of CKD (Table 6). The measure was translated into 68 languages to facilitate its use in global clinical trials. A conceptual framework for the final questionnaire is presented in Fig. 2. The questionnaire contains 8 items that assess the frequency of each symptom, all rated on a 5-point verbal rating scale. An additional 8 items assess the severity of these symptoms using an 11-point numerical rating scale. Five items assess the ability to do various activities, and 2 items assess the emotional impact of anemia of CKD.

**Table 6:** Content of the CKD-AQ

Symptom/Impact	Number of Items
**Frequency and severity**^**a**^	**14**
Very tired	2
Low energy	2
Weak	2
Chest pain	2
Shortness of breath during activity	2
Shortness of breath while at rest	2
Difficulty concentrating	2
**Severity**^**a**^	**1**
Bruised skin (past month recall period)	1
**Frequency**^**a**^	**1**
Difficulty remembering things	1
**Impact/ability to do activities**^**b**^	**5**
Standing for long periods of time	1
Sleeping	1
Didn’t want to do anything	1
Need to take a break	1
Need to take a nap	1
**Emotional impact**^**c**^	**2**
Distress	1
Feel burdensome	1

^a^The frequency of each symptom was rated on a 5-point Verbal Rating Scale ranging from “None of the time” to “All of the time.” Severity was rated on a 0–10 Numerical Rating Scale, with anchors of “0 = Absent/I did not have” to “10 = Worst Imaginable”

^b^Response options for ability to do various activities ranged from “None” to “A great deal”

^c^Response options ranged from “None of the time” to “All of the time”

## Discussion

To our knowledge, the CKD-AQ is the first PRO measure specific to patients with anemia of CKD developed using input from patients with anemia of CKD and in accordance with current best practices [6, 9, 10]. A review of the literature identified other PRO measures as potentially relevant for broader anemia populations; however, their applicability specifically to patients with anemia of CKD has not been demonstrated. Further, it is not clear if the Kidney Disease Quality of Life Instrument (KDQOL) and the Dialysis Symptom Inventory (DSI) questionnaires, developed for the CKD population, focused on symptoms of both anemia and CKD during the development process and therefore if all concepts would apply to the population with anemia of CKD.

The CKD-AQ was developed after collecting high-quality data in a rigorous manner directly from patients with anemia of CKD and provides insight on patients’ perspectives of symptoms and impacts on their daily lives. Frequency and severity of common symptoms were assessed, as well as the appropriate recall period for relevant symptoms. The patient-centered approach utilized to develop the CKD-AQ is an important feature because it ensures that the symptoms most relevant to patients with anemia of CKD were included. Moreover, although many of the symptoms and effects on HRQoL identified in our study are included in other PRO measures, to our knowledge, no other measures include assessments of both severity and frequency of all the relevant symptoms and impacts identified together in a single questionnaire (which can increase understanding of how patients experience symptoms and is recommended by FDA best-practices [9]). Other PRO measures also include additional concepts not relevant to patients with CKD.

Key findings from the qualitative CE interviews include that patients with anemia of CKD experience a wide range of symptoms that do not differ substantially based on a patient’s Hb concentration or whether the patient is on dialysis. However, symptoms classified as affecting daily life, being bothersome, or difficult to manage differed among patients, highlighting that not all patients experience symptoms in the same way. Some concepts, such as difficulty remembering things, difficulty concentrating, and restless legs, were most frequently reported only after probing. The need for probing highlights that patients may not spontaneously mention all applicable symptoms of anemia of CKD (as determined through review of the literature and clinician input), supporting the need for a dedicated PRO measure to assess and capture all symptoms patients experience that are potentially due to anemia of CKD. Additionally, our research found that attributing symptoms to CKD, anemia, or something else is very difficult for patients, and patients did not always know which of their symptoms were due to anemia of CKD. To ensure that the CKD-AQ includes the full range of potential symptoms of anemia of CKD, all concepts (whether attributed to anemia by patients or not) were reviewed by the research team for relevance to anemia of CKD prior to inclusion in the CKD-AQ, and the survey was designed so that attribution of the symptom to anemia was not necessary.

The content validity of the CKD-AQ was assessed through 3 rounds of interviews with patients with anemia of CKD. Data from these interviews helped ensure that the instructions and content of the measure were clear and accurately reflected the symptoms and impacts most important to patients with anemia of CKD. The CKD-AQ was reviewed and refined to reduce the overlap of concepts with common generic PRO measures, including the SF-36 and EQ-5D. This reduced the length of the questionnaire to facilitate its use in the clinical setting, reducing patient burden and allowing its possible use in conjunction with generic PRO measures without redundancy. Finally, a 7-day recall period was adopted for all questions in the PRO measure (except for bruising) for increased practicality and appropriate patient recall.

Anemia is a frequent complication in patients with CKD and, when untreated, the consequences can include impaired cognitive and cardiovascular function and accelerated progression of CKD [18]. As such, tools like the CKD-AQ that assess the frequency, severity, and impact on daily activities of symptoms of anemia of CKD have the potential to improve patients’ HRQoL.

Although the study was conducted according to best practices, there were a number of limitations. First, not all questions were asked of all participants because interview time was limited. However, not all concepts were relevant to every participant, especially if certain symptoms were not initially reported. Additionally, while we recruited a diverse population to obtain input on the symptoms of anemia of CKD across a range of patient characteristics, most participants were being treated for anemia. The burden for patients earlier in their disease course (not being treated for anemia) could be different or less noticed. Furthermore, all participants were recruited from large US dialysis centers and may not be representative of the entire US population affected by anemia of CKD or of patients of other countries. The large percentage of participants with comorbid conditions could also affect the results because symptoms of another condition could be attributed to anemia of CKD, and study participants often did not attribute their symptoms to anemia; however, this is a real-world population where patients will often have multiple comorbidities and care was taken to focus on the anemia-related symptoms and impacts.

After developing the questionnaire, linguistic translation and cultural adaptation into 68 languages were completed to facilitate use of the PRO measure in future global clinical studies. The questionnaire is being administered to thousands of patients in global trials (NCT03029208 and NCT02876835). The psychometric properties and scoring of the CKD-AQ will also be established based on results from these trials.

## Conclusion

In conclusion, this study reports the development of a novel PRO measure using a patient-centered approach in accordance with current best practices. The CKD-AQ captures the frequency and severity of the most relevant symptoms and impacts associated with anemia of CKD. It has the potential to assist clinicians in assessing and understanding patients’ symptoms due to anemia of CKD as well as to help evaluate treatments for anemia of CKD in clinical trials.

## Supplementary information

**Additional file 1: Item S1.** Research Team Characteristics and Relationship with Participants. **Table S1.** Research Team Characteristics. **Item S2.** Representative CE Interview Questions. **Item S3.** Literature Review Methods. **Table S2.** Detailed Search Strings for Selected CKD and Anemia PRO Measures.

## Data Availability

The datasets used and/or analyzed during the current study are available from the corresponding author on reasonable request.

## Abbreviations

CD: Cognitive debriefing
CE: Concept elicitation
CKD: Chronic kidney disease
CKD-AQ: CKD and Anemia Questionnaire
DSI: Dialysis Symptom Inventory
EMA: European Medicines Agency
EQ-5D: EuroQol-5D
ESA: Erythropoietin-stimulating agents
FDA: US Food and Drug Administration
GI: Gastrointestinal
Hb: Hemoglobin
HRQoL: Health-related quality of life
HS: High school
ISPOR: International Society of Pharmacoeconomic and Outcomes Research
KDQOL: Kidney Disease Quality of Life Instrument
PRO: Patient-reported outcome
SD: Standard deviation
SF-36: 36-Item Short Form Survey
