# Supplementary material for: Symptoms and impacts in anemia of chronic kidney disease
Source: J Patient Rep Outcomes. 2020 Jul 29;4:64. doi: 10.1186/s41687-020-00215-8 (PMC7391458; doi:10.1186/s41687-020-00215-8)
Supplement: Supplementary file 1 — Additional file 1: Item S1. Research Team Characteristics and Relationship with Participants. Table S1. Research Team Characteristics. Item S2. Representative CE Interview Questions. Item S3. Literature Review Methods. Table S2. Detailed Search Strings for Selected CKD and Anemia PRO Measures. [file 41687_2020_215_MOESM1_ESM.docx]

**SUPPLEMENTARY MATERIALS**

**Item S1: Research Team Characteristics and Relationship with Participants**

In-person, one-on-one interviews were conducted in a manner so that questions and answers were inaudible to others, if in the room, at either the dialysis center or nephrology clinic. Participants did not have a relationship with the interviewers prior to study commencement. Participants were told that the interviewers were from Health Outcomes Solutions and were conducting interviews with people with chronic kidney disease and anemia across the United States to learn more about patient symptoms and how having anemia and chronic kidney disease affects patients’ daily lives. Notes were taken during interviews as a backup if audio recording failed. Interviewees did not review the transcripts.

Characteristics of the research team are provided in Table S1.

**Table S1. Research Team Characteristics**

| **Name** | **Credentials** | **Occupation** | **Gender** | **Experience and Training** |
| --- | --- | --- | --- | --- |
| **Interviewers** | | | | |
| Susan D. Mathias | MPH | Health services researcher | Female | 30+ years of relevant experience conducting interviews, developing PRO measures, and analyzing and interpreting PRO data |
| Hilary H. Colwell | MPH | Health services researcher | Female | 20+ years of relevant experience conducting interviews, developing PRO measures, and analyzing and interpreting PRO data |
| Anne McCoy | JD | Health services researcher | Female | 10+ years of relevant experience conducting interviews |
| **Coders** | | | | |
| Hilary H. Colwell | MPH | Health services researcher | Female | See above |
| Anne McCoy | JD | Health services researcher | Female | See above |
| **Clinical Expert** | | | | |
| Kirsten L. Johansen | MD | Nephrologist | Female | Nephrology Division Director Co-Director, Chronic Disease Research Group, Hennepin County Medical Center |

**Item S2: Representative CE Interview Questions**

| - *Can you tell me what is it like to live with chronic kidney disease and anemia?* - *What bothers you most about chronic kidney disease and anemia?* - *What symptoms, if any, have you ever experienced as a result of your anemia associated with your CKD? [Probes: feeling tired/run down, not being able to concentrate, not being able to remember things?]* - *Are you able to know which symptoms are due to your chronic kidney disease, which are due to your anemia, or which are due to something else? [If yes] Which symptoms are due to chronic kidney disease and which are due to anemia?* - *Can you describe each of these symptoms to me? [Probes: onset, duration, severity, amount of limitation to daily activities?]* - *How does having anemia or chronic kidney disease affect your day-to-day life [Probes: ability to accomplish the things you’d like to do each day, work, school, childcare, chores at home, playing sports, avoiding activities?]* - *What is the worst thing about having anemia? Why? What about chronic kidney disease? Why?* |
| --- |

**Item S3: Literature Review Methods**

**Objectives:**

- To identify existing chronic kidney disease (CKD) and anemia-specific patient reported outcome (PRO) measures
- To identify concepts to explore during subsequent qualitative interviews with patients

**PubMed Search:**

The initial search was limited to publications available in English from January 2006 to March 2016 and conducted in PubMed using the following search string:

(CKD OR "Chronic Kidney Disease") AND (symptoms OR anemia OR fatigue OR energy OR cognition OR memory) AND questionnaire

**Identification of Candidate Measures for Further Review:**

The initial PubMed search yielded a total of 373 abstracts. Each abstract was reviewed to identify the PRO measures mentioned and the number of times each was mentioned. Of the 54 PRO measures identified, the majority were excluded from further review due to infrequent utilization (only appearing in 1-2 abstracts), because they were generic measures (i.e., SF-36, Patient Health Questionnaire-9, or EQ-5D), or because the topic of the measure was not deemed a relevant symptom of anemia of CKD (e.g., depression).

Two PRO measures, The Kidney Disease Quality of Life Instrument (KDQOL) and the Functional Assessment of Cancer Therapy – Anemia (FACT-An), were selected for further review based on relevance and high frequency of appearance in the abstracts identified in the initial search. Three additional PRO measures, The Fatigue Assessment Scale (FAS), Patient-Reported Outcome Measurement Information System Fatigue (PROMIS Fatigue), and Dialysis Symptom Inventory (DSI) were selected for an abbreviated review based on potential relevance.

Detailed searches for each of the 5 PRO measures selected for further review were then undertaken. A search, not limited by date, was conducted of the full name and/or abbreviation of the PRO measure using the search strings listed in Table S1. Articles identified through these searches that described either the development of the PRO or the evaluation of its measurement properties were selected for additional review. The 3 most recent clinical studies that included the measure were also reviewed.

**Table S2. Detailed Search Strings for Selected CKD and Anemia PRO Measures**

| **PRO Measure** | **Search String** |
| --- | --- |
| Kidney Disease Quality of Life Instrument (KDOQOL) | KDQOL OR KDQOL-SF OR KDQOL-36 OR "Kidney Disease Quality of Life Instrument" |
| Functional Assessment of Cancer Therapy – Anemia (FACT-An) | FACT-An OR FACT-Anemia |
| Fatigue Assessment Scale (FAS) | "fatigue assessment scale" |
| Patient-Reported Outcome Measurement Information System (PROMIS) Fatigue | PROMIS AND fatigue |
| Dialysis Symptom Inventory (DSI) | "dialysis symptom index" |

Potential shortcomings of the identified measures for use in patients with anemia of CKD include the following:

- The KDQOL assesses health-related quality of life, and while it does assess symptoms, it only assesses bother associated with symptoms rather than frequency and/or severity. The KDQOL was developed based on interviews with 13 patients with CKD, but it is not clear whether any of these patients had anemia of CKD, and it is not documented if saturation of concepts was demonstrated. In addition, its test-retest reliability has not been evaluated, nor has the amount of change that would be clinically meaningful (minimum important difference [MID]) been estimated for this measure.
- The FACT-An was developed based on interviews with 14 subjects with cancer and anemia, so it is unclear if it would be content valid for use in individuals with anemia of CKD. In addition, its responsiveness has not been evaluated. Finally, the FACT-An has not been utilized in any anemia of CKD studies, so it is unknown how it would perform.
- The FAS was developed based on existing fatigue scales without patient input. It has not been utilized in anemia of CKD studies, and some of its measurement properties have not been evaluated (known groups validity and responsiveness) nor has its MID been established.
- The PROMIS Fatigue was developed with patient input, but the sample size and clinical characteristics are not known. This measure has not been utilized in any anemia of CKD studies.
- The DSI was developed based on interviews with 13 individuals receiving dialysis. The only measurement property that has been evaluated is test-retest reliability, and it has not been utilized frequently.
